# Climate change and socioeconomic determinants are structural constraints to agency in diet-related non-communicable disease prevention in Vanuatu: a qualitative study

**DOI:** 10.1186/s12889-021-11245-2

**Published:** 2021-06-26

**Authors:** Amy Savage, Hilary Bambrick, Lachlan McIver, Danielle Gallegos

**Affiliations:** 1 School of Public Health & Social Work, Queensland University of Technology, Brisbane, Australia; 2 College of Medicine and Dentistry, James Cook University, Townsville, Queensland Australia; 3 Director Woolworths Centre for Child Nutrition Research, Queensland University of Technology, Brisbane, Australia; 4 School of Exercise & Nutrition Sciences, Queensland University of Technology, Brisbane, Australia

**Keywords:** Non-communicable diseases, Nutrition, Food security, Nutrition transition, Climate change, Qualitative

## Abstract

**Background:**

Pacific Island countries, many of which are low- and middle-income countries, have some of the highest rates of diet-related non-communicable diseases (DR-NCDs) globally. These countries also face some of the earliest and most significant impacts of climate change. Several pathways between climate change and DR-NCDs have been described in the literature; however, the scope is broad and lacks context specificity. This paper uses a case study of one Pacific Island country, Vanuatu, to investigate links between climate change and DR-NCDs.

**Methods:**

An ethnographic qualitative research approach was used to share the lived experiences of community participants and to explore and contrast these with the perspectives of key informants at the national level. Data collection comprised thirty-two semi-structured interviews and community fieldwork in two villages using a mix of methods, including group workshops, informal conversations, and observations. Reflexive thematic analysis was conducted on both data sets.

**Results:**

This study found that DR-NCDs are a prominent health concern for ni-Vanuatu people and that structural determinants, including climate change, are the main driving forces for increased DR-NCD risk in the country. However, there was a lack of understanding of the links between climate change and DR-NCDs both at the community and national levels. Structural factors, such as social determinants and climate change, constrained individual and community agency in making optimal food and health choices and promoted the nutrition transition in Vanuatu. Despite the critical role of social determinants and climate change in driving DR-NCD risk, the responsibility for prevention and treatment was considered to rest mainly with the individual. A systems approach is advocated to grasp the complexity and interrelatedness of the causes of DR-NCD risk.

**Conclusions:**

The interaction of structural determinants creates food and health environments that amplify the risk, burden, and consequences of DR-NCDs. It is recommended that the DR-NCD narrative in Vanuatu be re-framed with an emphasis on the range of structural determinants of DR-NCD risk. This will serve to enhance individual and collective agency to not only make healthy food and other behavioural choices but also to exercise agency to transform the structures in a culturally appropriate way.

**Supplementary Information:**

The online version contains supplementary material available at 10.1186/s12889-021-11245-2.

## Background

Non-communicable diseases (NCDs) are one of the most significant global health challenges of this century. The World Health Organization (WHO) estimates that approximately 41 million people die annually as a result of NCDs and 15 million of these are people aged between 30 and 69 years - considered premature deaths [1]. Three of the four NCDs that contribute to the most premature NCD deaths globally are diet-related NCDs (DR-NCDs); namely cardiovascular diseases (CVD), diabetes and some cancers [1]. Pacific Island countries (PICs), many of which are low- and middle-income countries (LMICs), have some of the highest rates of NCDs and this increasing burden is considered a regional “human, social and economic crisis” [2, 3]. Additionally, these countries are facing some of the earliest and most significant impacts of climate change globally [4]. This paper uses a case study of one PIC, Vanuatu, to investigate links between DR-NCDs and climate change.

NCD prevention has predominantly focussed on modifiable behavioural risk factors with an emphasis on reducing tobacco use, consuming healthy diets, increasing physical activity and limiting alcohol use [1]. As a result, NCDs have been commonly characterised as ‘lifestyle diseases’, with a strong onus on individual choice and responsibility [5, 6]. However, it has become increasingly apparent that structural determinants of health, including socioeconomic and environmental factors, represent significant causal factors in NCD risk [1, 5, 7]. Furthermore, these structural determinants shape, and often undermine, the ability of people to make healthy choices [8, 9].

The interplay of structure and agency in the social sciences, and applied to health promotion, is not new and the emphasis on one or the other, and their interaction, has long been debated [8]. Agency can be seen simply as the capability to act and often refers to an individual, however, it can also be applied to other actors, such as communities, corporations, or governments [9]. Structure is the framework or environment within which agency is exercised; it comprises the rules and resources (or sources of power) [8]. It is also crucial for health promotion to understand that agency can also be exercised to reinforce, reproduce or to transform existing structures, both intentionally and unintentionally [8, 10].

Anthony Giddens’ structuration theory highlighted the mutual construction of structure and agency whereby agents’ actions influence societal structures, and agency is influenced by those structures [11, 12]. The conflation of structure and agency in Giddens’ theory was criticized by some scholars, including Margaret Archer and critical realists more broadly, who built on structuration theory by proposing that structure and agency are indeed interdependent, however, they are considered to be analytically discrete [12, 13]. The reinforcement, reproduction, and transformation of structures takes place over time and existing structures are stable and affect current agency [13]. Bhaskar explains: “agents are always acting in a world of structural constraints and possibilities that they did not produce”, however, agents reproduce and transform those very structures through their activities and decisions [14 p. xvi]. The critical realist understanding of structure and agency is applied in this study, exploring the structural drivers of DR-NCDs and how they play out at the community level. Recognising and targeting these drivers has the potential not only to address a significant public health issue, but also to enhance the opportunities for people and communities to exercise individual and collective agency in health choices and the agency to shape those very structures.

Structural drivers of DR-NCDs include social, commercial and environmental trends and changes. The distribution of risk factors, morbidity, and mortality of NCDs are strongly influenced by social determinants of health that give rise to health inequalities [15]. Marmot and Bell describe social determinants as “the unequal conditions in which people are born, grow, live, work and age; and the inequities in power, money and resources that give rise to them” [15 p. 10]. Social determinants also shape the distribution of effects of other structural drivers of NCDs. For example, climate change disproportionately affects vulnerable populations who lack the resources to adequately respond [16, 17]. Recently, commercial determinants of health, and their role in driving NCDs, have also been emphasised [18, 19]. These commercial structural drivers of NCDs have been described as “strategies and approaches used by the private sector to promote products and choices that are detrimental to health” [18 p. e895]. Global environmental changes are also of increasing importance but have not been well incorporated in the NCD narrative [7]. Climate change, biodiversity loss, air pollution, land use and changes to agriculture have been identified as fundamental causes of increased NCD risk [6, 7]. In this paper, we explore the links between climate change and NCDs and the interactions with other structural drivers.

Several pathways between DR-NCDs and climate change have been previously described. A review by Frumkin and Haines [7] highlighted numerous links between climate change and NCDs. It was found that climate-induced disasters, resulting from increasing frequency and intensity of extreme weather events, adversely affect the ability of people with NCDs to access the required health services. Increased temperatures and extremely hot days lead to increased mortality and hospital admissions due to CVD and other NCDs, CVD risk due to sleep disturbance, and decreased physical activity which is associated with increased risk of CVD and some cancers. Furthermore, drinking water salinisation from sea level rise is likely to lead to increased hypertension. Climate change will also affect nutritional pathways of NCDs through decreased agricultural yields and the nutritional quality of food [7].

A review focused on the Pacific region found that climate change is compromising food and nutrition security (FNS) in a way that increases NCD risk through climate-related disasters, climate-induced migration and reduced agriculture and fisheries production [20]. In Vanuatu, it was found that climate change increases NCD risk by undermining FNS and exacerbating the nutrition transition and reliance on imported food products [21]. Additionally, a recent publication by *The Lancet* described the ‘global syndemic of obesity, undernutrition and climate change’ and emphasised the common underlying drivers [6]. Not only do these three pandemics have shared drivers, but the authors also note that “[t] he challenges facing action on obesity, undernutrition, and climate change are closely aligned with each other” [6 p. 792].

Although these reviews outline some of the critical pathways and connections between climate change and NCDs, the scope of the literature is broad and lacks context specificity. Few studies use primary data collection or explore context-specific links and potential interventions, or reflect local resilience, vulnerabilities and adaptive capacity [22]. This paper aims to contribute to addressing this gap with a case study on one PIC, Vanuatu, by exploring the potential causes of the increasing NCD burden in Vanuatu and the impacts of climate change on NCD risk. We investigate these topics using the perspectives and experiences of ni-Vanuatu (‘of Vanuatu’) in two villages and of health, development and climate change practitioners working in the country. This study interrogated the interaction of structure and agency in driving NCD risk and investigated how individuals and communities can exercise agency in respect of food and health choices.

## Methods

### Study setting

**Fig. 1:**
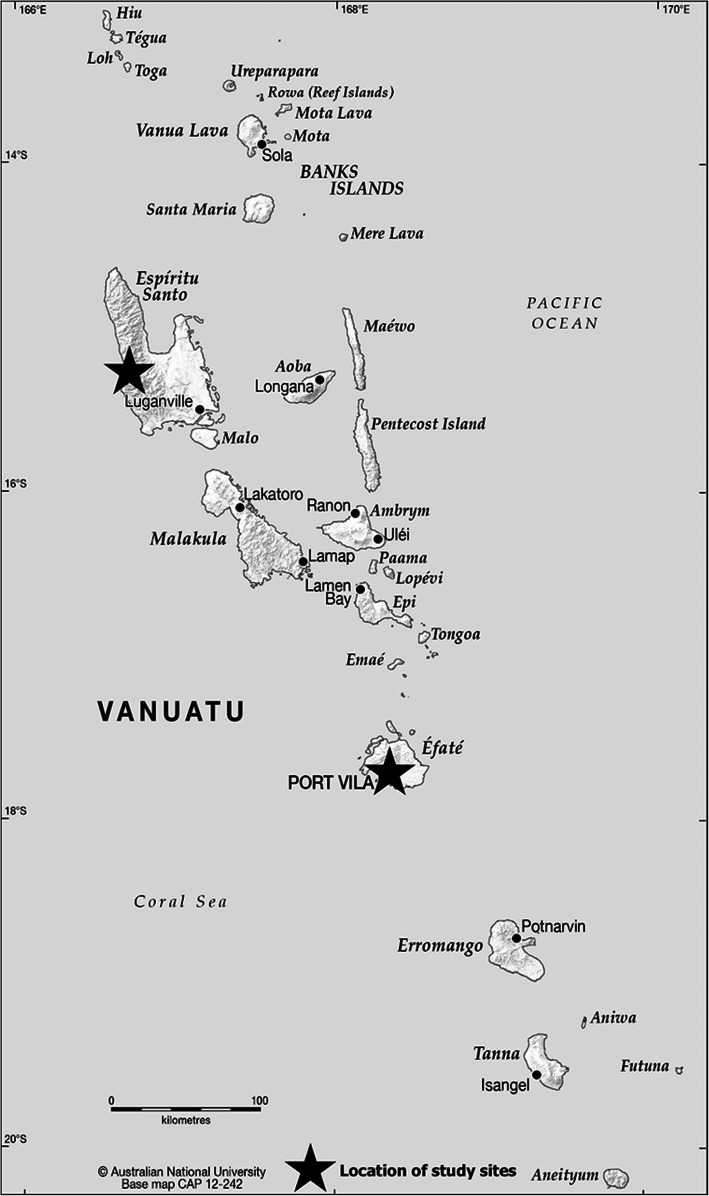
Map of Vanuatu Source: CartoGIS, The Australian National University [64]

Vanuatu is an archipelago of 83 islands, with a population of approximately 270,000 dispersed across the 65 inhabited islands (see Fig. 1) [23]. An estimated 75% of the population live in rural areas, which includes the peri-urban population [23]. The monthly per-capita income is USD160 in urban areas and USD140 in rural areas, and food expenditure is approximately 56% of total household income [24]. Vanuatu experiences a triple burden of malnutrition, whereby undernutrition, overweight and obesity and micronutrient deficiencies co-exist [25, 26]. Additionally, the country faces an increasingly high NCD burden with a high prevalence of DR-NCD risk factors including, hypertension, hyperglycaemia, and raised cholesterol [27]. Increasing NCDs are driven by the nutrition transition – a dietary shift away from traditional, locally grown foods to a greater dependence on imported foods higher in salt, sugar and fats [21, 26, 28].

Vanuatu has high exposure to natural hazards, including climate extremes such as tropical cyclones and drought. A global ranking has rated Vanuatu the most vulnerable country to natural disasters [29]. In the last five years, two Category 5 cyclones, the highest on the intensity scale, have devastated parts of the country [30, 31]. Vanuatu is already experiencing the effects of climate change, such as increased temperatures, sea-level rise and ocean acidification [32]. Furthermore, climate change is expected to further increase temperatures, sea-level rise and ocean acidification and result in more extremely hot days, coral reef bleaching, extreme rainfall days, and more intense, less frequent, tropical cyclones [32]. It is also projected that extreme El Niño-South Oscillation (ENSO) events, related to drought conditions in the South Western Pacific, will increase in frequency as a result of climate change [33, 34].

### Study design and data collection

This study forms part of a project investigating the impacts of climate change on FNS and DR-NCDs in Vanuatu employing a qualitative, ethnographic approach grounded in the public health discipline and underpinned by a critical realist ontology and epistemology. The study design aimed to collect and share the lived experiences of community participants and to explore and contrast these with the perspectives and practice of key informants at the national level. All data collection was conducted by the lead author as part of a PhD program at the Queensland University of Technology. She holds a Master of Human Nutrition, is proficient in Bislama, the national language of Vanuatu, and has significant professional experience working in cross-cultural settings, including extended periods of time in Vanuatu since 2013. A 10-week scoping visit was conducted in early 2017 to develop the data collection tools, build relationships with key stakeholders, and organize gatekeeper access.

**Fig. 2:**
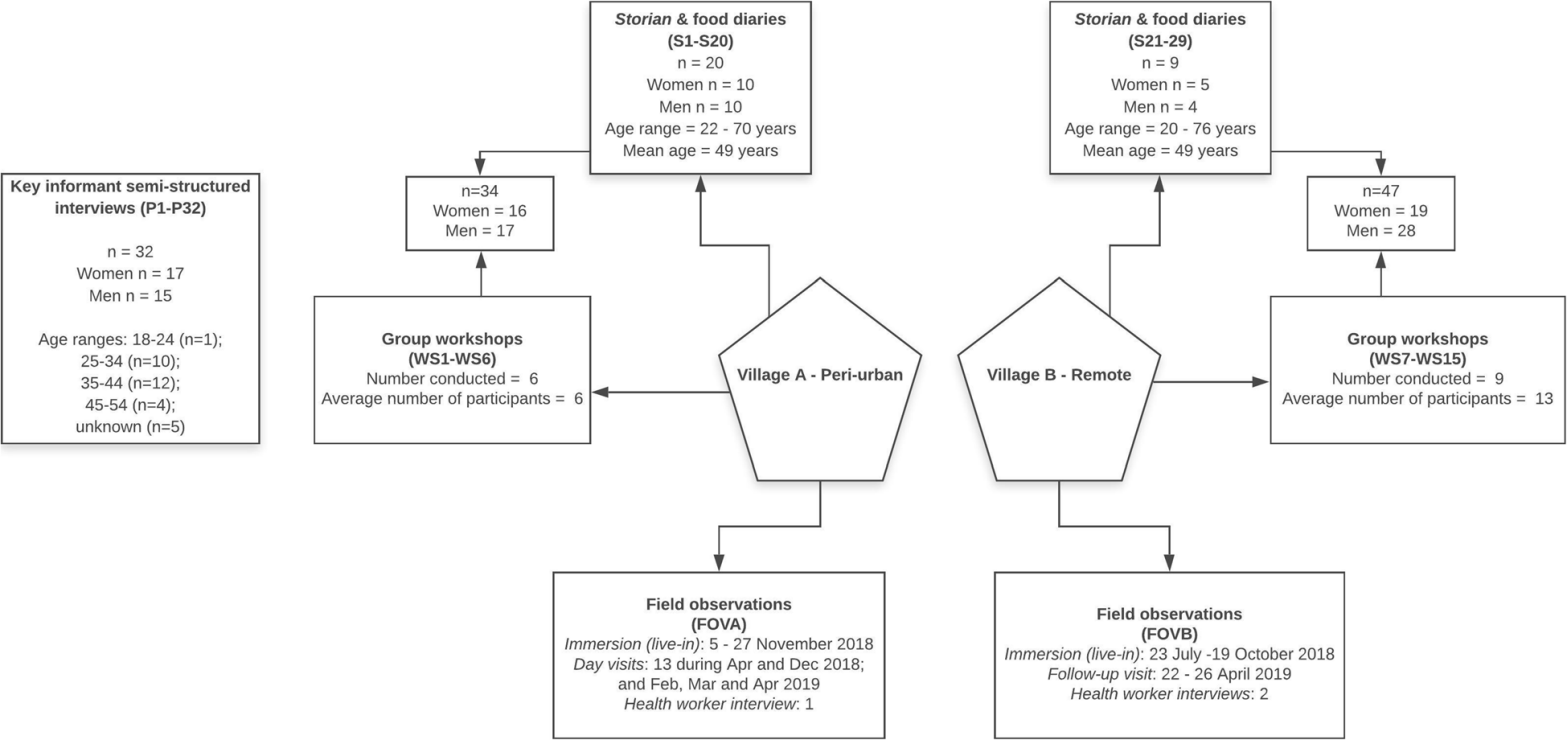
Data collection summary Source: adapted from [21]

Reflexivity, the ongoing process of critical reflection on the position of the researcher(s) in development of knowledge and the influence of their biases and worldviews on the interpretation of results, was a crucial aspect of data collection and analysis. The differences in the perspectives of the researchers and the participants were recognised and interrogated during all phases of the research project. A range of data collection methods was employed, including semi-structured interviews with key informants and community-based fieldwork. Each of the methods is elaborated below and summarised in Fig. 2.

#### Key stakeholder interviews

Thirty-two key informant interviews were conducted face-to-face, usually in the participants’ workplace or a public venue such as a café, in Port Vila (*n* = 26) and Luganville (n = 2) or via Skype (*n* = 4) between April 2018 and May 2019. Purposive snowball sampling was used to identify practitioners and experts in climate change, nutrition, food security and health and participants were recruited via email [35]. Participants did not have specific expertise in the connections between climate change, FNS and DR-NCDS. However, they had knowledge in relevant fields which are broadly categorised as climate change (*n* = 17), health (*n* = 15) and other (*n* = 3), (which includes migration, development, agriculture). Interviewees represented eight nationalities, including 16 (50%) ni-Vanuatu, and various organisations (see Table 1).

**Table 1:** Key informant interviews

Organisation type	Participants
Multilateral agencies	P4, P9, P10, P11, P17, P20, P25
Bilateral donor	P5, P23
Government of Vanuatu	P1, P2, P3, P16, P27, P29, P30, P32
Independent consultant	P12, P14, P19, P31
University/research institute	P18
Nongovernmental organisation	P6, P7, P8, P13, P15, P21, P22, P24, P26, P28

A semi-structured interview technique was employed to accommodate the varied areas of expertise, put participants at ease with a more conversational flow, reduce interviewer bias and allow for unexpected data. Interview durations ranged from approximately 25 min to 75mins and repeat interviews were not conducted. An interview guide was used, however, the conversations deviated from the guide depending on the interviewee’s experience. Interviews were conducted until it was determined by the lead author that data saturation had occurred – ie. when little or no new information was elicited with each new participant and no new themes emerged [36]. All interviews were conducted in English, recorded and transcribed professionally or by the lead author.

#### Community fieldwork

Community data collection was conducted in two villages: a peri-urban village on the outskirts of Port Vila (Village A) and a remote village on the west coast of Espiritu Santo (see Fig. 1). The two villages were selected purposively as representing a remote and a peri-urban village and because of gatekeeper access obtained through local organizations. Key features of each village are described in Table 2. Data was collected using various qualitative methods to gather lived experiences at the community level. The lead author resided in Village A for three weeks in November 2018 and made day visits during 2018 and 2019, and in Village B for ten weeks during July – October 2018. Field notes were recorded daily and included personal reflections and observations of conversations, interactions and local practices, with an emphasis on food-related practices, hierarchies, institutions and culture. *Storian,* which can be simply translated as ‘to story’ from Bislama, were conducted. These were informal, one-on-one (or sometimes with the assistance of a fieldworker) conversations of approximately 45–90 min. The format of *storian* drew on a life history approach, encouraging participants to tell stories about their lives, from childhood to the present [37]. The interviewer, while encouraging an informal approach with free dialogue, directed the conversations to stories about food, agriculture, climate change and related topics.

**Table 2:** Summary of study sites, reproduced from [21]

	Village A	Village B
**Island**	Efate	Espiritu Santo
**Village type**	Peri-urban	Remote
**Population**	5211 people 965 households	136 people 37 households
**Closest city**	Port Vila, capital city	Luganville
**Access to city**	Port Vila centre: 20–30 min’ drive Regular local buses Cost: USD1.30 each way	Luganville: 3–4 h truck ride on unpaved roads; and 2–4 h boat trip Private chartered transport Cost: approximately USD165.00 each way
**Electricity access**	Municipal electricity	No electricity supply Small solar lights One solar inverter
**Water supply**	Municipal water supply	Gravity-fed from river
**Main source of household income**	Wages/salary	Sale of fish/crops/handicrafts
**Main cooking method**	Gas stove Open fire inside an outdoor kitchen/near the house	Open fire inside a hut (kitchen)/outside near the house
**Food storage**	Very few refrigerators – can rent space in a refrigerator/freezer Dry storage – no preservation	Traditional storage e.g. yam beds Harvest as needed

In each village, group workshops were conducted based on the Climate Change & Food Security Vulnerability Assessment guide [38]. Workshops included: a transect walk; village map; historical timeline; wellbeing ranking; livelihoods strategies and seasonal calendar; climate risk ranking and coping mechanisms matrix; food systems diagram; and institutional mapping. Each workshop was facilitated by the lead author and a local research assistant over a duration of two to six hours, depending on the content and group engagement. Workshop activity outputs and researcher notes were produced for each session.

Data collection methods varied between the two study villages. In Village A, it was difficult to recruit participants for group workshops; however, people were enthusiastic about participating in *storian*. Additionally, a shorter residential stay and ongoing day visits were more feasible in the peri-urban setting. In Village B, on the other hand, engagement in group workshops was high, but people were less receptive to individual *storian*. An extended residential stay was necessary due to the remote location. As a result, in Village A more *storian* data was collected, while in Village B, there was more observational and workshop data. The majority of community fieldwork was conducted in Bislama, in which the lead author is proficient, with the assistance of local fieldworkers. Community data was translated into English by the lead author.

#### Ethics

The Human Research Ethics Committees of the University of Queensland (2017001510) and Queensland University of Technology (1900000071) provided ethics approvals. Research approval was obtained from the Vanuatu National Cultural Council, including a research visa, and support letters were provided by the Ministry of Health and Ministry of Agriculture, Livestock, Forestry, Fisheries and Biosecurity. Participants were provided a plain language statement, in either Bislama or English, and the purpose of the study was discussed before and during research activities. Informed written consent was obtained for interviews, *storian* and group workshop participation. Village chiefs, and the elders in Village B, provided written consent for observational data collection.

### Data analysis

QSR NVivo software was used for data management and as a tool for data coding [39]. Reflexive thematic analysis was conducted on both sets of data, starting with a familiarisation process and open, inductive coding by the lead author (AS) [40, 41]. A subset of the data, approximately 30%, was independently coded by one co-author (DG). Codes were then re-organised and grouped to identify higher-level concepts, and AS and DG regularly discussed theme development. These discussions contributed to theme co-development that interrogated individual frames of reference. All authors agreed on final themes and analysis. Quotes are used throughout the chapter to present participants’ voices. A unique code identifies participants based on method type: interviews (P1, P2...P32); *storian* (S1, S2 … S29); group workshops (WS1 … WS15); field observations are identified by village (FOVA/FOVB).

## Results

The analysis showed that participants were concerned about DR-NCDs and attributed the increasing burden primarily to changing diets and, to a lesser extent, reduced physical activity. Structural determinants and constraints to agency were evident as primary drivers of DR-NCDs. In particular, social determinants such as urbanisation, poverty and changing ways of life, along with environmental factors, including climate change, were found to be critical drivers of a changing diet and physical activity patterns. Additionally, the health system had limited capacity to promote DR-NCD prevention in an effective and culturally supportive way. Despite the drivers of DR-NCDs identified in the data being firmly rooted in deeper structures and processes, they were often framed as individual choices or shortcomings with an emphasis on individual responsibility for prevention. Finally, it was found that a systems approach was useful to grasp the complexity and interrelatedness of the causes of the nutrition transition and increased DR-NCD risk. These five key themes are described in more detail in the sections below.

### The high and increasing burden of DR-NCDs as a health concern

Awareness and concern for the burden of NCDs were common to all participants. One participant stated: “*Everybody is aware of this NCD crisis …* “ (P3). Participants commonly linked the causation of DR-NCDs to a changing diet comprising of a higher proportion of ‘store-bought’ foods, as well as decreased physical activity and broad changing ways of life.

Type 2 diabetes, or *‘sik suga’*, was the most commonly discussed NCD, and many participants shared their experiences, or those of a family member, of diabetes-related amputations. Hypertension, CVD and obesity were also often mentioned along with, less frequently, mental health and some cancers. While NCD awareness was high in both villages, NCD experiences were more common in peri-urban Village A. Many participants had stories to share of NCDs affecting themselves or close family members, including in the younger population. The health clinic nurse stated that there were 22 registered cases (patients that seek treatment after a screening) of hypertension and 14 of diabetes; however, she noted that the figures are likely much higher as many people do not seek health services. The nurse noted that heart disease and cancer rates were low but said that it was because *“everyone is already dead from these”* (FOVA). Participants shared a diabetes-related amputation story of a young villager in their early twenties. The health clinic reported five diabetes-related amputees in the village. While the existence and consequences of DR-NCDs and many of their risk factors were common knowledge in the village, the translation of awareness to knowledge and action for prevention appeared to be low.

In contrast, in Village B a local nurse explained that DR-NCD rates in the region were low with only two cases of diabetes, one case of hypertension and some cases of overweight and obesity with *“less [cases] than town [Luganville]”* (FOVB). He said there was no heart disease in the region; however, when asked, he also confirmed that there was no screening or diagnosis of heart disease. The first diabetes-related amputation in the region occurred during fieldwork in July 2018, signalling a changing NCD risk profile in the area. One participant noted that more people were aware of diabetes because of the first amputation and, as a result, people were beginning to limit their intake of sugar.

### Structural constraints to agency in making health choices

The leading cause of DR-NCDs consistently mentioned by participants was a dietary shift away from locally grown foods to a greater reliance on imported foods. The store-bought foods mentioned by participants and observed comprised mainly of white rice, white bread, instant noodles, cooking oils and tinned fish and meats.

*“NCDs, particularly diabetes, is a big problem. This is the case in all of Vanuatu, and it’s because of the change in diet.”* (S13)

#### Social determinants: changing ways of life and the nutrition transition

**Fig. 3:**
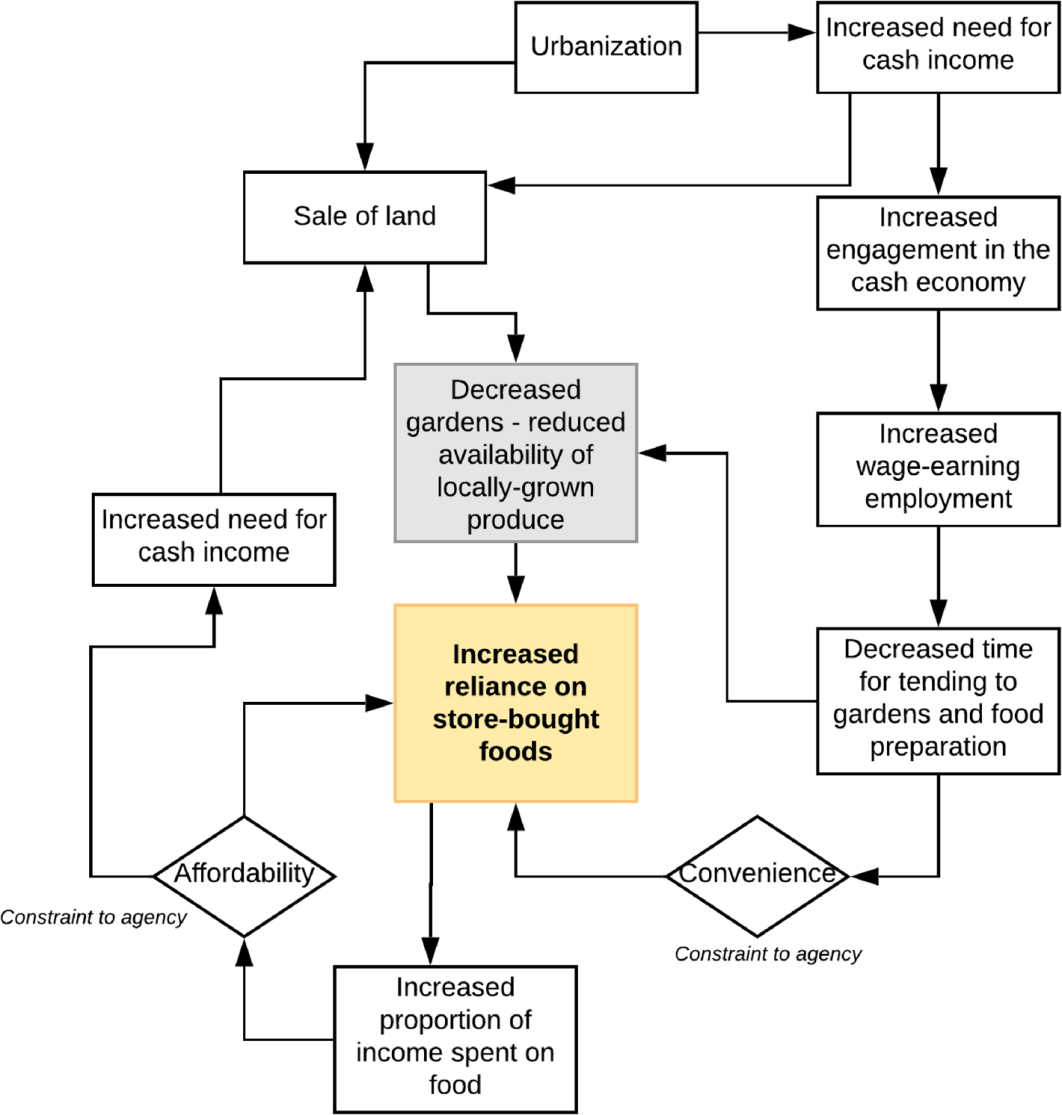
Social determinants, changing ways of life and the nutrition transition in Village A

A mutually reinforcing cycle of socioeconomic factors was found to be driving the nutrition transition and increasing DR-NCD burden in the peri-urban setting, Village A. In Village B, there were indications of the beginning of a similar trajectory; however, a subsistence way of life remained predominant. The cycle, outlined in Fig. 3, was perceived as a general change in ways of life and manifested as a social trend of urbanisation and increased engagement in the cash economy. These changes led to an increased need for income to support increasing expenses, such as school fees and electricity. As more people participated in wage-earning employment, including more women, there was less time for tending to gardens and food preparation. As a result, people increasingly relied on *convenient* store-bought foods which increased the proportion of income spent on food, thus increasing the need for a cash income. This then increased the reliance on store-bought foods that were *cheaper* than locally grown produce, again reinforcing the dependence on imported foods.

Furthermore, people sold more land for income generation, and in response to increasing urbanisation and migration, which reduced the availability of land for food production. Reduced gardening resulted in a reduction in traditional forms of physical activity and the availability of locally grown foods. The interrelated socioeconomic trends and determinants have restricted the ability of people to choose foods based on health or cultural preferences:

*“ … It’s changed lifestyles...because the urbanisation of almost all districts is growing and people are moving to towns, and you go to town, the food there is cheaper, the imported food - eating rice is probably cheaper than buying food, and one of the factors: local foods are expensive.”* (P32)

*“ … people know that food is connected to health and which foods are healthy, but they don’t have a choice. They must eat, and local food is expensive.”* (S12)

Participants also described dietary changes occurring in rural villages. A health worker posted near Village B expressed concern that DR-NCD rates will increase in the future in the region:

*“I am worried that this place will go in the direction of town, but I don’t want that to happen.”* (FOVB)

#### Climate change and DR-NCDs

There was little awareness of the impacts of climate change on NCDs, or on health more broadly. Key informants provided occasional instances of connecting climate change and NCDs including: the physiological impacts of heat stress and extreme temperatures; the impacts on the health system and its ability to manage people with NCDs; reduced physical activity; and the underlying similarities between climate change and NCDs as complex problems with long lag times in the lead-up to impacts, that require systems-level responses. Additionally, links between climate change and dietary change, including reduced quality, were often made. While awareness of DR-NCDs and climate change as isolated issues was ubiquitous, there was a disconnect in linking them. Overall, few participants were aware of, or could articulate, the links between climate change and DR-NCDs. Some participants were aware of this lack of awareness, for example:

*“ … probably from my interview, you can tell I haven’t thought about it enough yet, and many people probably haven’t.”* (P4)

Awareness of the links between climate change and DR-NCDs was also obscured by the increasing general awareness of climate change in Vanuatu, but often flawed understanding. In Vanuatu, ‘climate change’ was commonly used as an explanation for many unrelated changes and shocks. One participant suggested this might also be the case for NCDs:

*“Why are more people getting more diabetes?” “Oh, climate change.” … So, climate change is being attributed to everything at the moment.”* (P5)

Although explicit connections between climate change and DR-NCDs were not well described, and at the community level direct links between climate change and DR-NCDs were not identified, participants discussed the impacts of climate change on agriculture and FNS leading to sub-optimal nutritional outcomes. Participants highlighted the effects of climate change on reduced diet quality. Difficulties with changing seasons, such as prolonged, or more frequent, dry or wet periods, and the adverse effects of this on local agriculture were discussed. Decreased availability of local crops, mainly fresh fruits and vegetables, resulting from climate impacts was linked to an increased dependence on imported, store-bought foods leading to a lower quality diet and increased risk of DR-NCDs.

*“ … obviously we want people to eat more local crops … more fruits and vegetables, less processed food. But if those are not practically available, then they’re going to choose what is available, and what is affordable, and that’s not going to be the healthier option. So, we’re going to see worsening of dietary patterns and, that in turn, will contribute to NCDs.”* (P11)

Participants highlighted the long-term effects of increased frequency and intensity of climate extremes on the diet. Climate extremes, such as cyclones and drought, destroyed gardens and impaired short-term FNS, but coping and adaptation strategies also undermined long-term FNS and contributed to dietary change. For example, in Village A, people described increasing the proportions of store-bought foods in their diet, even after cyclone recovery. They attributed this to increased prices of locally grown produce and the provision of food aid following tropical cyclones. Additionally, frequent climate extremes were described as a barrier for continuing subsistence gardening in Village A where store-bought foods were cheap and readily available. One participant described the increased dependence on store-bought foods following climate extremes:

*“If we have more cyclones coming up, it will destroy gardening, and when you’re lazy to do gardening or lazy to do what you’ve learned from … some of the experts, then you will continue to buy things in shops.”* (P23)

The remoteness of Village B seemed to be a protective factor in maintaining a more traditional diet, even in the face of climate extremes. Villagers described increasing their rice consumption because of changing food preferences following food aid after TC Nigel in the 1980s and increased food purchases during the 2015–2017 El Niño drought. However, relative to Village A, the ongoing contribution of store-bought foods in the diet was minimal.

Participants also noted that climate change, particularly climate extremes such as heatwaves, will have adverse effects for those with pre-existing NCDs. Furthermore, it was mentioned that climate-induced disasters will negatively impact an already overburdened health system:

*“To me, what happens when we have a disaster, or an event … we have additional demands placed on the health system. The health system struggles to deal with that, to the point that they have to divert attention to it, and other things in the health system stop.”* (P4)

### Barriers to health service access

Barriers to health service access for NCDs and an emphasis on treatment rather than prevention were noted as impediments to adequately addressing the NCD burden in Vanuatu. Barriers included cultural differences in health-seeking behaviours, lack of health and nutrition literacy, physical inaccessibility and the cost of services. Health service access was observed to be low in both villages. Participants described having a limited capacity for early recognition of NCD symptoms and often accessed health services at advanced stages of disease progression. One interviewee described health-seeking behaviours:

*“ … there’s a perception, and I’ve heard it many, many times, that the hospital is the place that you go to die. And the feedback loop is there. People don’t understand symptoms. By the time they get to the hospital they’re at death’s door … “* (P12)

One participant expressed the impacts of low health service access:

*“My wife now has both legs amputated from diabetes. Before she got her legs amputated, she didn’t know she had diabetes – she never went to the hospital.”* (S16)

An individual responsibility approach tended to be the focus of NCD prevention. However, health and nutrition literacy was perceived to be low, and several participants discussed the lack of clear messages around NCD prevention or the ability to apply recommendations practically. Some participants described hearing conflicting advice from nurses and health workers about nutrition and NCD prevention which they considered confusing for patients:

*“Because they are kind of making them confused, even they’re stopping them from eating local foods...Because there’s no nutrition training provided to these nurses, on how to counsel and what exactly to tell them.”* (P3)

Participants explained that they had learned about diabetes from friends and relatives and that they expected further education from the government to make informed health and food choices. The nurses at the health clinic in Village A noted that they recommend that people increase exercise and consume more fruits and vegetables and *aelan kakae* (island food). However, many participants were not sure how to effectively implement this advice, either due to not being confident in their nutrition knowledge, or because of other constraints, including financial and cultural. Traditional nutritional knowledge has been informally passed down through generations; however, this knowledge is being undermined by new, imported foods and socioeconomic and climate pressures changing the diet, along with changes in ways of life, disconnecting people from the practices of sharing traditional knowledge.

A key challenge in addressing NCDs in Vanuatu noted by participants was the tendency for both national-level, and individual-level, response to emphasise treatment rather than prevention.

*“ … we go on NCD screening... whenever we find somebody ill, then we have to send them over for further management. Yeah that’s all. We send them for treatment, not with something like counselling. So, we’re sending them for medication.”* (P3)

This is likely partially due to the limited capacity, and particularly a lack of resources, both human and financial, to address the increasing NCD burden in the country. Participants noted that there were insufficient resources to test for diet-related health issues, such as micronutrient deficiencies or cholesterol, and there was only one nutritionist in the Ministry of Health and one NCD-qualified nurse in Port Vila. For example:

*“As the end result, we are seeing more complications in diabetes. When you go to provinces, you see it more evident, but the main point is because we have limited human resource capacity, we cannot really work with the people when they are high risk.”* (P10)

In both villages, sickness and death were also often explained as being caused by *kastom*^1^ or black magic. The use of traditional healers practising *kastom* medicine were evident. In Village B, consultations with traditional healers were often the first action taken in addressing a health issue, with these practitioners commonly called upon to treat physical ailments believed to be caused by black magic. In Village A, participants explained that *kastom* medicine has a ‘good side’ and a ‘bad side’: traditional healing and black magic. One participant who is a double leg amputee described her struggle with diabetes and mentioned her amputations in the context of diabetes. However, she also explained that:

*“ … it wasn’t really diabetes – it was kastom to cut off my leg.”* (S15)

Additionally, Vanuatu is a strongly Christian country, and religious beliefs may sometimes be a hindrance to seeking health services and applying prevention measures for NCDs. Some people believed that faith in God and Christian healers can heal sickness. It was common for health issues to be explained by religious-related causes, black magic, contemporary medical explanations or a mix of all three. For example, the cause of a stillborn child in Village B was explained both as a punishment from God because of disputes in the family and because it was a breech birth.

### Individual responsibility

The onus on individual responsibility in DR-NCD prevention was evident in discussions related to changing ways of life and implications for health. Ni-Vanuatu participants frequently described ‘laziness’ in relation to increasing DR-NCDs. It was discussed in the context of people being too lazy to work in the gardens because it is much easier to earn money and buy food. For example, one interviewee explained:

*“Some is laziness, some just looking...to get money to sit around and just buy from the shop, no need to plant.”* (P31)

*Laez* in Bislama is not an easy concept to translate and represent. It is likely derived from the English word *lazy*, and it can be used in a similar context, but it can also mean something akin to ‘cannot be bothered’, but often with less negative connotation than in English. Laez was used to describe changing diets intertwined with changing ways of life and loss of *kastom* and traditional knowledge. It was more commonly used in the peri-urban setting than in the rural village. One participant describes:

*“There are a lot of people that don’t work, but they also don’t make a garden. There are big changes to the culture. The cost of living has changed, and the culture is being lost. People want to adopt a different culture. So they don’t make a garden.”* (WS5)

The explanation that people are ‘lazy’ was commonly used to explain why individuals do not choose to eat a healthier diet to prevent DR-NCDs. For example, local health workers expressed frustration when recommendations are not followed:

*“They say that eating only, or mostly, aelan kakae [island food] is possible. They just want food from the store. They are lazy. They eat two-minute noodles, tinned meat. Kids eat dry noodles at school. But they know [the health risks]. But to change the diet is hard.”* (FOVA)

Although less common in Village B, *laez* seemed to be an emerging concern. For example, one participant described a conversation between some ‘young people’ that he overheard:

*“ … if they had enough money they would just buy rice and food from the store – it is easier, and they would be too lazy to go work in the gardens. I think that this is not good – it is good to work in the gardens for exercise as well as good food.”* (FOVB)

Both the nurses in Village B also noted difficulties for individual behaviour change in dietary practices; however, it was attributed to a lack of understanding or a tendency to *“continue with the same practices”* rather than laziness (FOVB). The emphasis on individual responsibility is contradictory to the traditional collectivist culture, including the outlook on food and health, which was strongly apparent in the rural setting. Tradition, along with seasonality, were the main observed drivers of food choice. When asked about how people make food choices, one participant described:

*“ … people don’t consciously think about their food choices. They just do what they grow up with or what everyone else is doing and don’t really think about it.” (FOVB)*

The emphasis on individual responsibility was also visible in the way villagers described dietary and physical activity behaviours, usually to address diagnosed DR-NCDs, such as:

*“I have changed what I eat, but sometimes it is hard. I was about 60 years old when I found out about my high blood pressure.”* (S10)

One participant described:

*“Some people do know what is not good in their diet, but they still want to have it. For example, sometimes single amputees become double amputees because they don’t behave.”* (S13)

### Complexity and feedbacks

**Fig. 4:**
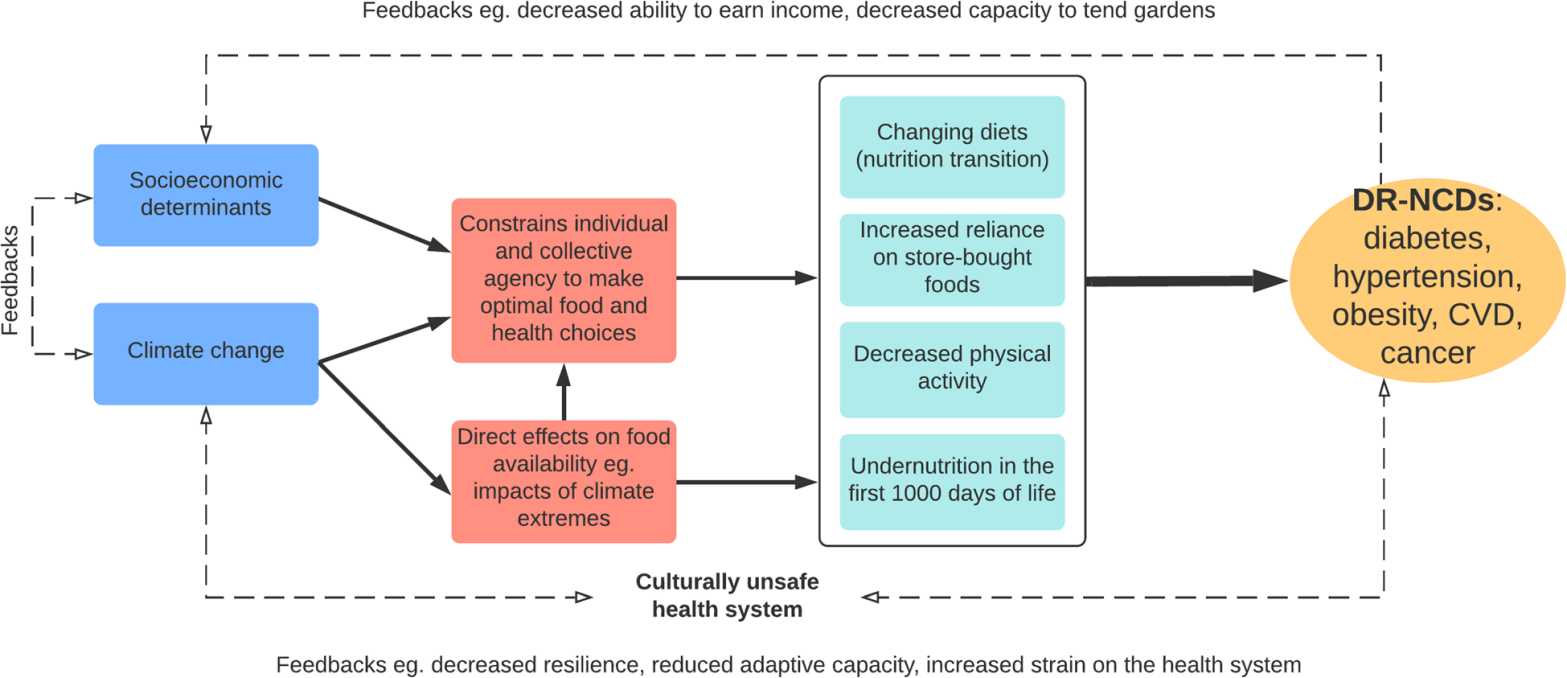
Drivers of NCDs in Vanuatu: complexity and feedback loops

The causes of increased DR-NCDs observed in this study were not linear and straightforward but characterised by complexity and feedbacks. Figure 4 provides a visual depiction of the relationships between the key themes. Climate change directly impairs FNS leading to increased risk of DR-NCDs via adverse effects on agriculture and fisheries. Socioeconomic factors undermine climate change resilience and adaptive capacity exacerbating the effects of climate change by limiting the coping and response strategies available to people. For example, people with low incomes, in the face of reduced crop yields or destroyed gardens, have little option but to purchase cheap food items which are often of poor dietary quality. Similarly, climate change exacerbates the socioeconomic determinants leading to dietary change and increased DR-NCD risk, and DR-NCDs. For example, climate change, in rendering subsistence gardening more difficult and less reliable, serves as a push factor for changing ways of life with a greater dependence on cash income and store-bought foods.

Some key informants mentioned this complexity. One interviewee described an example linking climate change, disease and impaired nutritional outcomes and how each relates to, and exacerbates, the other:

*“That’s another thing with climate change, isn’t it? There’s a lot of links that are two to three degrees of separation that if someone gets ill, it’s going to affect their nutritional [status] … , but vice versa, if they’re nutritionally compromised, they’re more likely to get ill … “* (P4)

Key informants discussed common challenges in addressing DR-NCDs and climate change, such as the extended lag times in the visible presentation of symptoms or impacts; their cross-sectoral nature; and the need to address from a systems perspective:

*“ … addressing them both is complicated … … But for NCDs and climate change, it really needs to be a system-wide approach … “* (P4)

*“ … we shouldn’t be just working in the symptoms, symptoms of disease or symptom of the system, we need to look at the system foundation otherwise, it will not work here, and that’s why this climate change is expanding now more.”* (P10)

## Discussion

The picture that emerges from the analysis above is that there was widespread awareness and concern about the increasing DR-NCD burden in Vanuatu, but that the responsibility for prevention and treatment largely remained with the individual. Structural factors, including socioeconomic determinants and climate change, were found to be the fundamental drivers of diet-related DR-NCDs in Vanuatu and this is, in part, because they constrain individual and community agency in making optimal choices for their health. A systems approach is therefore needed to understand the links between climate change and health and to identify double-duty or triple-duty responses that simultaneously address both challenges.

### Re-framing the DR-NCD narrative

The results showed the primary cause of DR-NCDs was understood to be a changing diet, commonly known in the literature as the ‘nutrition transition’ [43], which was found to be driven by a range of social determinants and exacerbated by climate change. What is striking is that although these underlying structural processes were found to be driving increased DR-NCD risk and constraining individual agency to make health-related choices, an emphasis on individual responsibility prevailed, in both the community-level understanding of DR-NCD prevention and among expert informants. The analysis showed a disconnect between what participants think needs to be done to address DR-NCDs, in particular personal dietary behaviours, and the options that are legitimately available to people.

Dominant NCD discourse puts the onus on the individual and suggests that NCDs arise primarily from poor ‘lifestyle’ choices. The importance placed on individual agency and personal behaviours in public health has arisen as a result of the prevailing neo-liberalist paradigm and individualist cultures. Rushton and Williams [44] state that “[p] art of the problem with many of the constructivist approaches to global health governance (and indeed to global governance more broadly) is the over-emphasis on agency and the neglect of deeper structural determinants” (p.162). During the colonial era in Vanuatu “[t] he discourse of health care practitioners reveals that blame for malnutrition lies with mothers, not with the larger structural problems with access to land for subsistence agriculture, clean water, or comprehensive biomedical health care, systemic problems that are much more difficult to fix” [45 p.106]. However, over recent decades there has been a wider push to shift the NCD narrative to emphasise the significance of structural determinants, such as socioeconomic determinants or commercial determinants of health [5, 9, 19]. Lee and Crosbie (2020 p.315) describe this “alternative perspective … [as] pushing against the notion that NCDs are primarily self-inflicted, and that people must simply be convinced of the error of their unhealthy ways”.

The main structural determinants of DR-NCD risk found in this study included socioeconomic factors, such as urbanisation, linked to a shift away from subsistence gardening; poverty, resulting in the cost of food as a driver of food choice; increased engagement in markets and cash economy and increasing participation of women in the workforce; changing land use leading to, again, decreased subsistence gardening; and climate change. The findings of this study position climate change as a structural determinant of health which influences agency and both affects, and is affected by, social structures. Critical realism perceives society and social events and activities as occurring in open, dynamic systems “in which any number of occurrences and events can overlap and interact and in which people can learn and change” [13, 46]. Climate change is one of multiple sub-systems which influence FNS and DR-NCD risk and health cannot be extricated from these overlapping structural influences; from economics, from politics, or from climate change. While socioeconomic factors and climate change are recognised structural determinants by the WHO [1, 47], our study showed that within the health system in Vanuatu there was an emphasis primarily on the treatment of NCDs and individual responsibility.

DR-NCD prevention focused on individual behaviour change – a narrative that was prevalent at both the community and individual level. Participants commonly highlighted the need for improved nutrition and health education and counselling in communities. While enhanced education would improve the agency of communities and individuals to make healthy choices, a focus on behaviour change for DR-NCD prevention appears very unlikely to prove effective in Vanuatu if the structural constraints to agency are not addressed.

The study also found that the health system did not adequately support agency in the prevention and treatment of DR-NCDs in other ways. The health system, borne out of colonialism and sustained by a neo-liberalist development agenda, emphasises NCD treatment rather than prevention and individual responsibility for NCD prevention. There are several significant barriers to health service access, including physical and financial access. However, the primary impediment appeared to be the misalignment of the ‘modern’ health system with traditional health practices. Traditional healers and medicine, religious practices, and the sharing of collective medical knowledge and methods within the community appeared to be the first step in addressing health issues, and *kastom* explanations of disease causation were common. There is literature that demonstrates the widespread use and importance of traditional health services and *kastom* explanations for the cause of a range of illnesses in Vanuatu [48–54]. File and McLaws [48] describe the health system in Vanuatu as comprising three ‘domains’ for health-seeking behaviours: firstly, traditional healers (which included Christian religious healers); secondly, community and family members; and thirdly, biomedical practitioners. It was also shown that villagers respected biomedical health services but would often only seek these services when the ailment was considered serious [48]. Maden et al. [52], and our study results, also indicate that many people present to the hospital or clinic only when the illness is severe, which is often too late.

Aspects of the colonially imposed, ‘Western’, health system in Vanuatu could be considered ‘culturally unsafe’ as culture are often not adequately considered in health-seeking behaviours. The term’ cultural safety’ refers not only to the recognition and acceptance of different cultural practices in health but considers how “historical, economical, and social contexts influence health status and health care services” and related power imbalances [55 p.152]. Actions which serve to discount and devalue cultural practices and identity are considered ‘unsafe’ – a concept that has been used to examine the colonial health systems in other countries, such as Australia, New Zealand, and Canada [56–58]. The three ‘domains’ of health services in Vanuatu have a role to play in the overarching health system. These do not need to be mutually exclusive and traditional and western health services need to be better integrated as part of building a culturally safe health system. File and McLaws [48] found that “communities are pragmatic and pluralistic, integrating new paradigms into their belief systems” (p.6) and that collaboration of biomedical practitioners with traditional healers offers opportunities for more effective health services. In Vanuatu, there is an apparent dichotomy between traditional health practices and ‘Western’ medicine, but also opportunities to better merge the two systems, both in services and education, for enhancing NCD prevention and treatment. The establishment of a culturally safe health system has potential to contribute to the re-framing of the narrative of DR-NCDs in Vanuatu and improve prevention and treatment by decreasing the barriers to health service access and building a more holistic and culturally inclusive approach to prevention.

### Climate change, systems thinking and syndemic theory

In this paper, climate change was shown to drive NCD risk factors through a complex interplay of direct effects on agriculture and exacerbation of socioeconomic determinants leading to limited individual and collective agency to make food and health choices. Through these mechanisms climate change contributes to an increasing reliance on store-bought foods, decreasing physical activity, and is further driving the nutrition transition. Furthermore, in Savage et al. [21], it was found that the effects of climate change led to restricted dietary intake and diversity, particularly in the remote village, which likely resulted in acute undernutrition in many villagers. Undernutrition in the first 1000 days of life can lead to irreversible impacts, such as stunting, and increased risk of DR-NCDs later in life [59, 60]. Figure 4 summarises the connections between climate change and increased DR-NCD risk.

Despite high awareness and concern for both climate change and NCDs by a majority of participants, both at the national level and in the villages, there was little awareness of the impact of climate change on NCDs. Relationships between climate change and NCDs were described by some key stakeholder participants. While community participants did not articulate links between climate change and NCDs, the connections were illustrated in the stories, observations and workshop engagement documented in the two villages. The community data demonstrate the effects of climate change on FNS exacerbating the nutrition transition and contributing to an increasing prevalence of DR-NCDs. A recent study in Barbados involving interviews with key health professionals showed similar findings [22]. The study found that although most participants identified NCDs or NCD risk factors as the most significant health challenge in the country, knowledge of the relationships between climate change and NCDs were not commonplace [22].

Although climate change is a significant structural determinant of health and NCDs, the lack of awareness is unsurprising. The literature on the impact of climate change on NCDs while growing, remains sparse, and the translation from academic literature to policy, practice and community awareness takes time. Frumkin and Haines [7] state that global environmental changes, including climate change, are “a set of causal factors whose importance is increasingly clear but largely overlooked to date … “ (p.262). The impacts of climate change on NCDs are not direct, nor easily defined and measured; there are long lag times for observable effects to present, both for climate change and NCDs; and there are many interacting influencing factors, which present significant challenges for research.

The lack of understanding of the links between climate change and NCDs is also reflected in the lack of in-depth understanding of each of these issues individually because they both require specific disciplinary expertise. For example, we found a high general *awareness* of climate change; however, an *understanding* of climate change was not as common, particularly at the community level. The attribution of climate change to unrelated events and circumstances risks real action due to complacency from overexposure. Likewise, the education and messaging around NCDs were found to be inadequate, and community members often lacked the knowledge, or as discussed above, the agency, to implement preventative strategies, within their individual and community context. Key informants’ knowledge also often did not sufficiently cross disciplinary boundaries to connect climate change and NCDs. At the national level, the inadequate knowledge of the connections between climate change and NCDs has significant policy implications. Springer et al. [22] noted: “It is troubling that plans to tackle the NCD crisis in Barbados and improve health outcomes have been discussed under the assumption of a stable climate” (p.198). An improved understanding of the connections between climate change and NCDs by policy- and decision-makers has the potential to not only better address these significant health challenges faced by communities across Vanuatu, but to do so in a coordinated way that builds resilience and adaptive capacity.

Systems thinking and syndemic theory have gained traction for understanding and addressing climate change and NCDs [5, 6, 61]. Syndemic theory recognises the interplay between diseases and health conditions, emphasises the prominent role of social and environmental conditions that give rise to the development, transmission and severity of disease, and “assess the disease within its encompassing biocultural environment” [61 p. 136]. A recent article termed obesity, undernutrition and climate change “the Global Syndemic” and applied a systems perspective to understand the underlying, shared drivers, of these three global epidemics [6]. It was found that the main systems underpinning the Global Syndemic are food and agriculture, transportation, urban design, and land use. The findings of our study show similar results, albeit at a much more localised level.

A systems perspective that integrates context-specific local experiences with the broader systems with which they interact, and within which they operate, has the potential for more effective and appropriate interventions that consider structural determinants, feedback loops and community realities. Recognising the syndemic of climate change and all forms of malnutrition highlights the interplay between disease and climate change and the common underlying drivers. For example, socioeconomic determinants also contribute to climate change vulnerability and influence resilience and adaptive capacity, thereby creating critical feedback loops. This was also highlighted in the study in Barbados, as one participant noted the same social determinants of health are also determinants of climate change vulnerability [22].

Importantly, a syndemic approach within a systems perspective allows the identification of ‘double-duty’ or ‘triple-duty’ actions: actions that address undernutrition, DR-NCDs (including obesity) and/or climate change [6, 62]. Swinburne et al. outline recommendations for double-duty and triple-duty actions to address the Global Syndemic at global, national and community governance levels [6]. However, a place-based approach to designing and implementing such actions is necessary to ensure that specific constraints to agency are identified and people, communities, and nations are empowered with the ability to shape the food system and make health and food choices. The structural determinants, their interactions, and their manifestations at the local level differs from region to region, country to country and even within countries, for example, between rural and urban settings. The results of our study, and further targeted, qualitative, local-level research, will be crucial for informing an effective and appropriate design of such actions.

### Strengths and limitations

This study had limitations that should be considered in the interpretation and application of the results. The research focussed on *diet-related* NCDs and all participants were aware of the study aims. Diabetes and hypertension were the two NCDs most commonly discussed by participants; however, other NCDs – whether pathophysiologically linked to diet and nutrition or not – may not have featured prominently due to potential bias related to the study focus on FNS and DR-NCDs. On the other hand, diabetes and hypertension are two of the most prevalent NCDs in the country, while noting that they are those for which screening is most common and practical in Vanuatu [63].

While each key informant possessed relevant expertise, this expertise was varied and for the majority of participants did not cover all three topic areas of climate change, FNS and DR-NCDs. Nevertheless, the siloed nature of the expertise in each sector highlights the need for enhanced integration of public health and climate change agendas to address these interrelated challenges.

The study was conducted in only two villages in Vanuatu – a linguistically, culturally and geographically diverse country. The study sites were, however, purposively selected for their specific geographical, socioeconomic and cultural characteristics to represent urban-rural populations and a range features common to ni-Vanuatu villages. Bislama was the primary language for community data collection; however, during discussions and everyday interactions, villagers used indigenous languages. While local field assistants were engaged to lessen this constraint, it is likely that not all potentially relevant data was captured during fieldwork.

Finally, this research was not exhaustive. Instead, it aimed to share the experiences of people in the two study villages and of the key informants and present a local perspective within the existing literature on climate change and NCDs. The local context is specific, and these findings may not be broadly generalisable. They do, however, address gaps and contribute to the broader narrative on the topic. The findings and implications are also likely applicable, to some extent, to other PICs, SIDS and developing countries with similar characteristics, such as small widely-dispersed populations; comparable traditional diets and farming methods; increasing urbanisation; and an ongoing dietary transition away from a locally grown traditional diet towards a diet high in energy-dense, nutrient-poor, foods.

## Conclusion

This study found that DR-NCDs are a prominent health concern for ni-Vanuatu people and that structural determinants, such as socioeconomic factors and climate change, are the main driving forces for increased DR-NCD risk in the country. The primary cause of DR-NCDs, as understood by participants, was the shift in dietary patterns from a diverse traditional diet high in locally grown produce to an increased reliance on a limited number of imported food products. This dietary shift appears to be intertwined with broader changes in ways of life and is perceived to be driven by structural socioeconomic determinants and climate change. Nevertheless, NCD prevention emphasised an individual responsibility to make personal healthy behavioural choices within a ‘Western’ model of health and disease. Furthermore, structural socioeconomic and environmental determinants significantly constrained collective and individual agency to make beneficial choices about food and health. The interaction of these structural determinants creates food and health environments that amplify the risk, burden and consequences of DR-NCDs.

It is recommended that the DR-NCD narrative in Vanuatu is re-framed with an emphasis on addressing the range of structural determinants – social, commercial and environmental – of DR-NCD risk. This will serve to enhance individual and collective agency to not only make healthy food and other behavioural choices but also to exercise agency to transform the structures in a culturally appropriate way. Plausible pathways between climate change and NCDs were evident in this study, and double-duty and triple-duty actions can address health risks and simultaneously build adaptive capacity and resilience to the effects of climate change. This study demonstrates that a systems approach is crucial to understand the interconnecting systems and shared drivers of NCDs and climate change. A systems perspective also has the potential to link context-specific local realities with broader system drivers and inform policy decisions with community-level experiences to ensure that strategies and interventions are targeted appropriately.

## Supplementary Information

**Additional file 1.**

## Data Availability

The datasets generated and/or analysed during the current study are not publicly available due to participant confidentiality but are available from the corresponding author on reasonable request.

## Abbreviations

CVD: cardiovascular disease
DR-NCDs: diet-related non-communicable diseases
FNS: food and nutrition security
LMICs: low- and middle-income countries
NCDs: non-communicable diseases
PICs: Pacific Island Countries
WHO: World Health Organization

## Footnotes

1 *Kastom* is not easily defined within a colonial worldview but can be understood the “aspects of our [ni-Vanuatu] culture and society that can be demonstrated to have their origins in the pre-European-contact era” [42, p.38].
