# Supplementary material for: Climate change and socioeconomic determinants are structural constraints to agency in diet-related non-communicable disease prevention in Vanuatu: a qualitative study
Source: BMC Public Health. 2021 Jun 26;21:1231. doi: 10.1186/s12889-021-11245-2 (PMC8235621; doi:10.1186/s12889-021-11245-2)
Supplement: Supplementary file 1 — Additional file 1. [file 12889_2021_11245_MOESM1_ESM.pdf]

Semi-structured Interviews – Key Stakeholders (Government, Donors, Multilaterals, NGOs)

In line with the Talanoa/Storian method of data collection these interviews will begin with relaxed conversation and an open question initiating the topic but allowing the participant to direct where the conversation goes. Below are some guiding questions.

Participant ID:
Nationality:
Country of Residence:
M/F
Profession/Area of work:
Age group: 18-24 / 25-34 / 35-44 / 45-54 / 55-64 / 65+

1. *What do you think are the key nutrition-related health issues in Vanuatu?/ Wanem yu ting bigfala issues blo nutrition blo Vanuatu?*

   (Further question if NCDs are not mentioned: Do you think NCDs such as obesity, cardiovascular disease, diabetes etc are a key concern for Vanuatu? / Yu ting NCDs olsem obesity, cardiovascular disease, diabetes etc bigfala issues blo Vanuatu?)

2. *Has the ni-vanuatu diet changed over the last 10-20 years? If so, how?/ Kakae blo Vanuatu hemi jenis lo pas 10-20 yia? Sipos yes, olsem wanem?*

3. *What do you think are the main drivers of these changes? / Yu ting from wanem kakae blo Vanuatu hemi jenis?*

4. *Do you think that the effects of climate change influence the food and nutrition security of Ni-Vanuatu and affect the aforementioned health concerns? / Yu ting se climate change kilim food and nutrition security blo ni-vanuatu mo helt blo pipol blo Vanuatu?*

   Possible extra question: What do you think is the biggest threat to food and nutrition security in Vanuatu? / Yu ting wanem se bigfala trabol blong food and nutrition security blong Vanuatu?

5. *Do you know of any climate change adaptation strategies or coping strategies that communities or households are employing in Vanuatu? How do you think these affect their food and nutrition security? / Yu save sam adaptation o coping strategy se community o household blong Vanuatu mekem lo climate change? Yu ting olsem wanem kilim food and nutrition security blong Vanuatu?*

6. *Is there anything else that you would like to add? / Yu wantem talem samting mo?*
